# Classic hodgkin lymphoma associated with Epstein–Barr virus reactivation during vedolizumab therapy for ulcerative colitis

**DOI:** 10.1007/s12328-026-02302-7

**Published:** 2026-02-28

**Authors:** Tatsuya Hasegawa, Masatsugu Kojima, Toru Miyake, Mai Noujima, Takayuki Imai, Soichiro Tani, Keiji Muramoto, Tomoharu Shimizu, Takuji Iwashita, Masaji Tani

**Affiliations:** 1 Department of Surgery, Shiga University of Medical Science, Tsukinowa-Cho, Seta, Otsu, Shiga 520 2192 Japan; 2 Department of Pathology, Shiga University of Medical Science, Tsukinowa-Cho, Seta, Otsu, Shiga Japan; 3 Department of Gastroenterology, Shiga University of Medical Science, Tsukinowa-Cho, Seta, Otsu, Shiga Japan; 4 Medical Safety Section, Shiga University of Medical Science Hospital, Tsukinowa-Cho, Seta, Otsu, Shiga Japan

**Keywords:** Ulcerative colitis, Epstein–Barr virus, Vedolizumab, Classic Hodgkin lymphoma, Intestinal malignant lymphoma

## Abstract

Epstein–Barr virus (EBV)–associated lymphoproliferative disorders have been reported in patients receiving immunosuppressive therapy for inflammatory bowel disease; however, the onset of classic Hodgkin lymphoma (cHL) during vedolizumab therapy is extremely rare. A 73 year-old woman with ulcerative colitis (UC) was steroid-dependent, showing disease exacerbation when prednisolone was tapered or discontinued. Azathioprine was administered eight years prior but was discontinued after a short period due to liver dysfunction. Vedolizumab was initiated 31 months before presentation. Despite initial improvements in UC with vedolizumab, hematochezia subsequently persisted. Other biologics or immunomodulators that could cause systemic immunosuppression were avoided because she was receiving treatment for breast cancer. Colonoscopy showed erosions in the transverse colon and an ulcerative lesion in the sigmoid colon. A biopsy revealed high-grade dysplasia in the transverse colon and inflammatory granulation tissue without malignancy in the sigmoid lesion; therefore, surgery was indicated. Laparoscopic total proctocolectomy was performed, and the surgical specimen contained Reed–Sternberg cells positive for CD20, CD30, PD-L1, and EBV-encoded RNA, establishing EBV-associated cHL. Although the development of cHL is likely multifactorial, clinicians should be aware that EBV-associated intestinal cHL may develop in patients with UC receiving vedolizumab when tumor-like ulcers or mural thickening are encountered.

## Introduction

Ulcerative colitis (UC) is a chronic inflammatory disease characterized by recurrent inflammation of the colorectal mucosa. It typically involves the rectum, extending proximally and continuously along the colon [1]. The global prevalence of UC is increasing, and geographic variations are observed worldwide [2]. Although the exact cause of UC remains unknown, genetic susceptibility, intestinal microbiota, intestinal immune responses, and environmental factors have been reported as contributing factors [3–5].

Immunomodulators and biological agents have dramatically improved UC management. However, prolonged immunosuppression with these medications may lead to reactivation of latent Epstein–Barr virus (EBV) infection, predisposing patients to lymphoproliferative disorders (LPDs) [6]. Vedolizumab is a gut-selective monoclonal antibody against α4β7 integrin that has recently been developed as a molecularly targeted therapeutic agent. It blocks lymphocyte trafficking and may be effective against UC [7]. Vedolizumab is thought to have minimal systemic immunosuppressive effects; however, intestinal EBV reactivation remains possible.

Herein, we report a rare case of EBV reactivation–associated classic Hodgkin lymphoma (cHL) that developed during vedolizumab therapy for UC.

## Case report

A 73-year-old woman was diagnosed with extensive-type UC 13 years before the current presentation. Previous treatments included prednisolone, 5-aminosalicylic acid, and azathioprine; azathioprine was discontinued after only two weeks of treatment, eight years prior to presentation, because of liver dysfunction. No other biological agents were administered. The patient was steroid-dependent, with disease exacerbation when prednisolone was tapered or discontinued.

Vedolizumab therapy was initiated 31 months before the current presentation. Fifteen months prior to presentation, she was diagnosed with breast cancer and underwent partial mastectomy, followed by adjuvant therapy with an aromatase inhibitor. Although her colitis symptoms temporarily improved with vedolizumab, hematochezia persisted; however, the use of other biological agents or immunomodulators that could cause systemic immunosuppression was considered difficult because of undergoing breast cancer treatment.

**Fig. 1:**
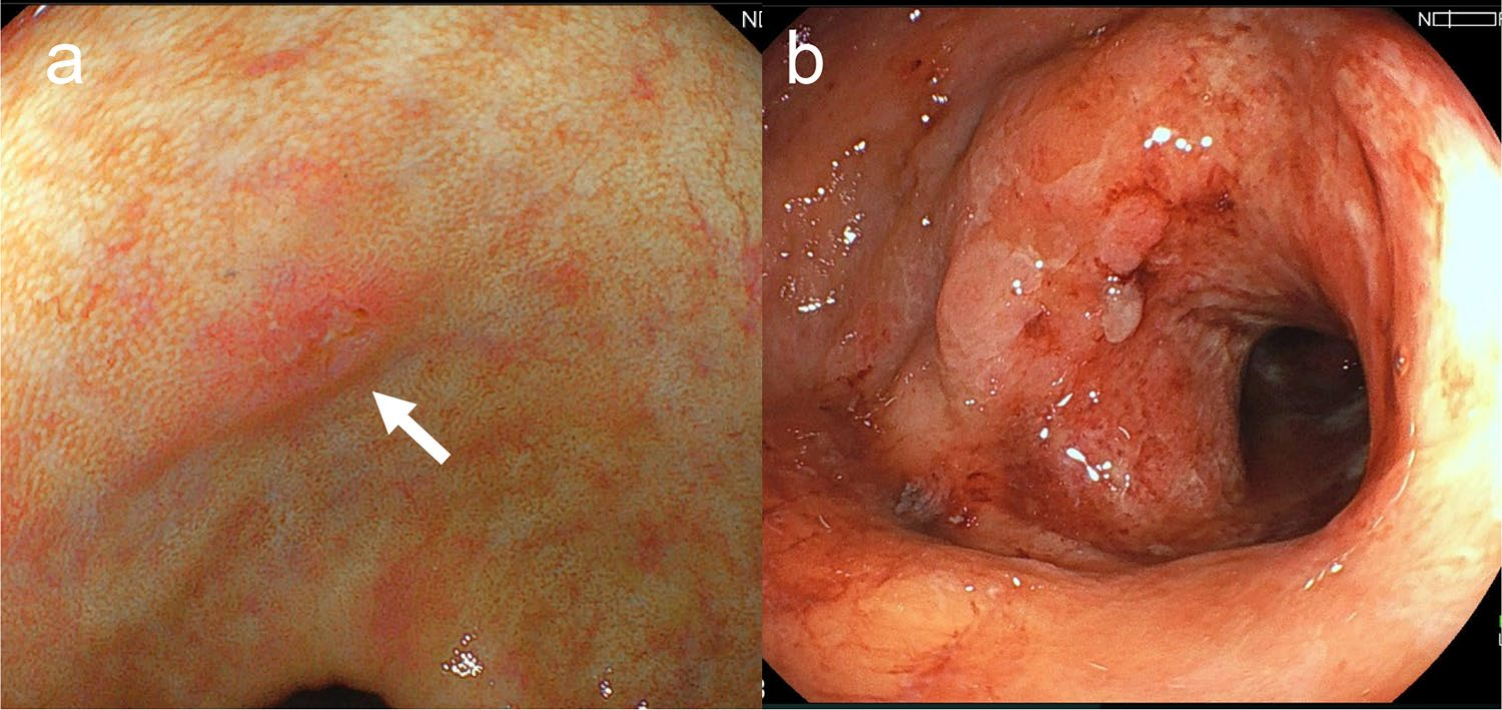
Preoperative endoscopic findings. **a** Irregular erosions in the transverse colon (arrow), with biopsy specimens showing high-grade dysplasia. **b** Circumferential ulcerative lesion with mural thickening in the sigmoid colon, with biopsy specimens showing inflammatory granulation tissue with no malignancy

**Fig. 2:**
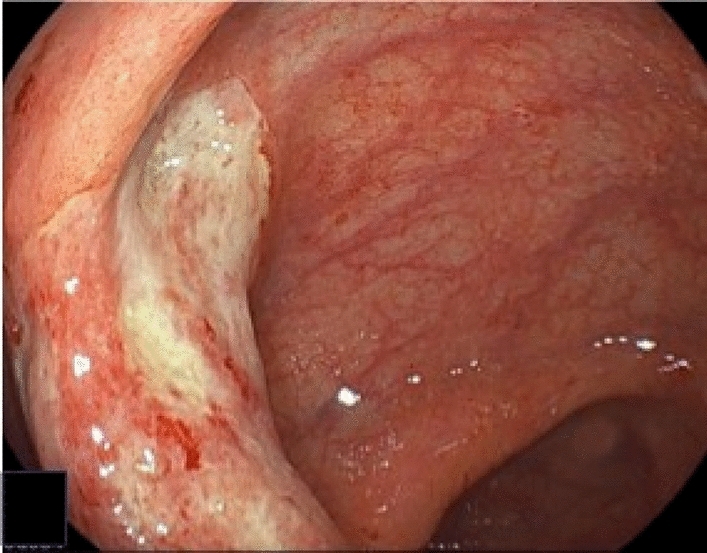
Endoscopic findings obtained 18 months before surgery. Endoscopy demonstrated　 a shallow ulcerative lesion with mild mural thickening involving approximately one-quarter of the colonic circumference, showing a morphology closer to the typical ulcerative appearance of gastrointestinal lymphoma

**Fig. 3:**
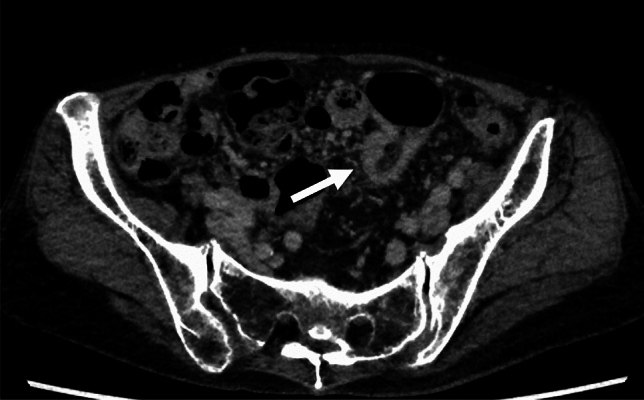
Contrast-enhanced computed tomography findings. Contrast-enhanced computed tomography revealing circumferential wall thickening of the sigmoid colon (arrow), raising the suspicion of a neoplastic lesion. No significant lymph node enlargement was observed elsewhere in the body, including the mesenteric regions

Colonoscopy showed irregular erosions in the transverse colon (Fig. 1a) and a circumferential ulcerative lesion with mural thickening in the sigmoid colon (Fig. 1b). Biopsy specimens revealed high-grade dysplasia in the transverse colon, whereas only inflammatory granulation tissue was identified in the sigmoid colon, with no malignant features detected. Eighteen months before surgery, endoscopy revealed a quarter-circumferential lesion with shallow wall thickening accompanied by ulceration in the sigmoid colon (Fig. 2). Histological examination of biopsy specimens obtained at that time demonstrated inflammatory granulation tissue, and no malignant features were detected, similar to the findings of the preoperative biopsy. Computed tomography (CT) revealed a circumferential wall-thickening lesion in the sigmoid colon, which was suspected to be malignant (Fig. 3). No significant lymph node enlargement was observed throughout the body, including in the mesenteric region.

**Fig. 4:**
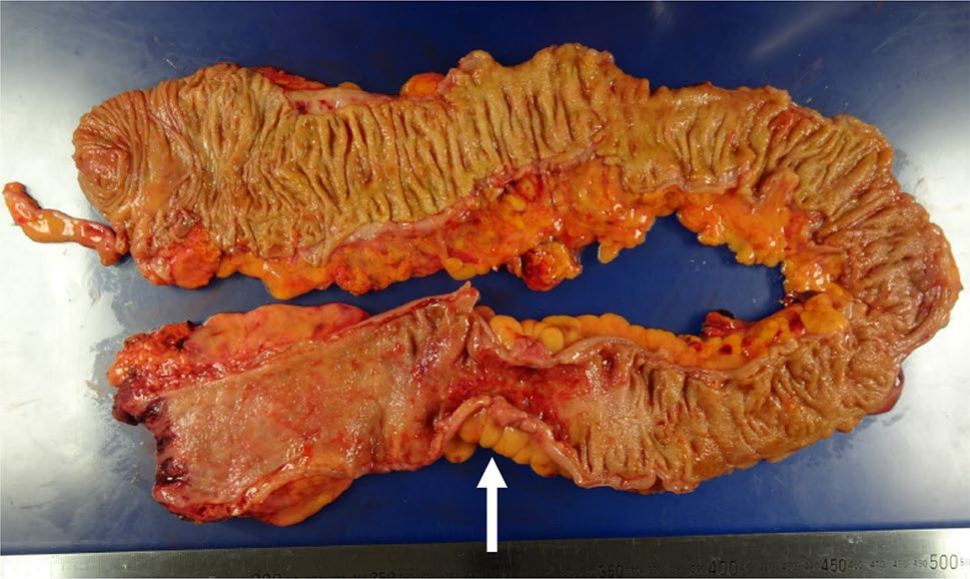
Macroscopic findings of the resected specimen. The resected specimen shows circumferential wall thickening in the sigmoid colon (arrow), suggesting a neoplastic lesion. No other apparent tumorous lesions were identified

Given the high-grade dysplasia in the transverse colon, the suspicion of malignancy in the sigmoid colon, and refractory hematochezia despite medical therapy, a decision was made to proceed with surgery. The patient underwent laparoscopic total proctocolectomy with an ileal pouch–anal anastomosis. The resected specimen is shown in Fig. 4. Circumferential wall thickening suggestive of a tumor was observed only in the sigmoid colon; no other tumorous lesions were identified. The operation was uneventful, and postoperative recovery was smooth. The patient was discharged on postoperative day 12.

**Fig. 5:**
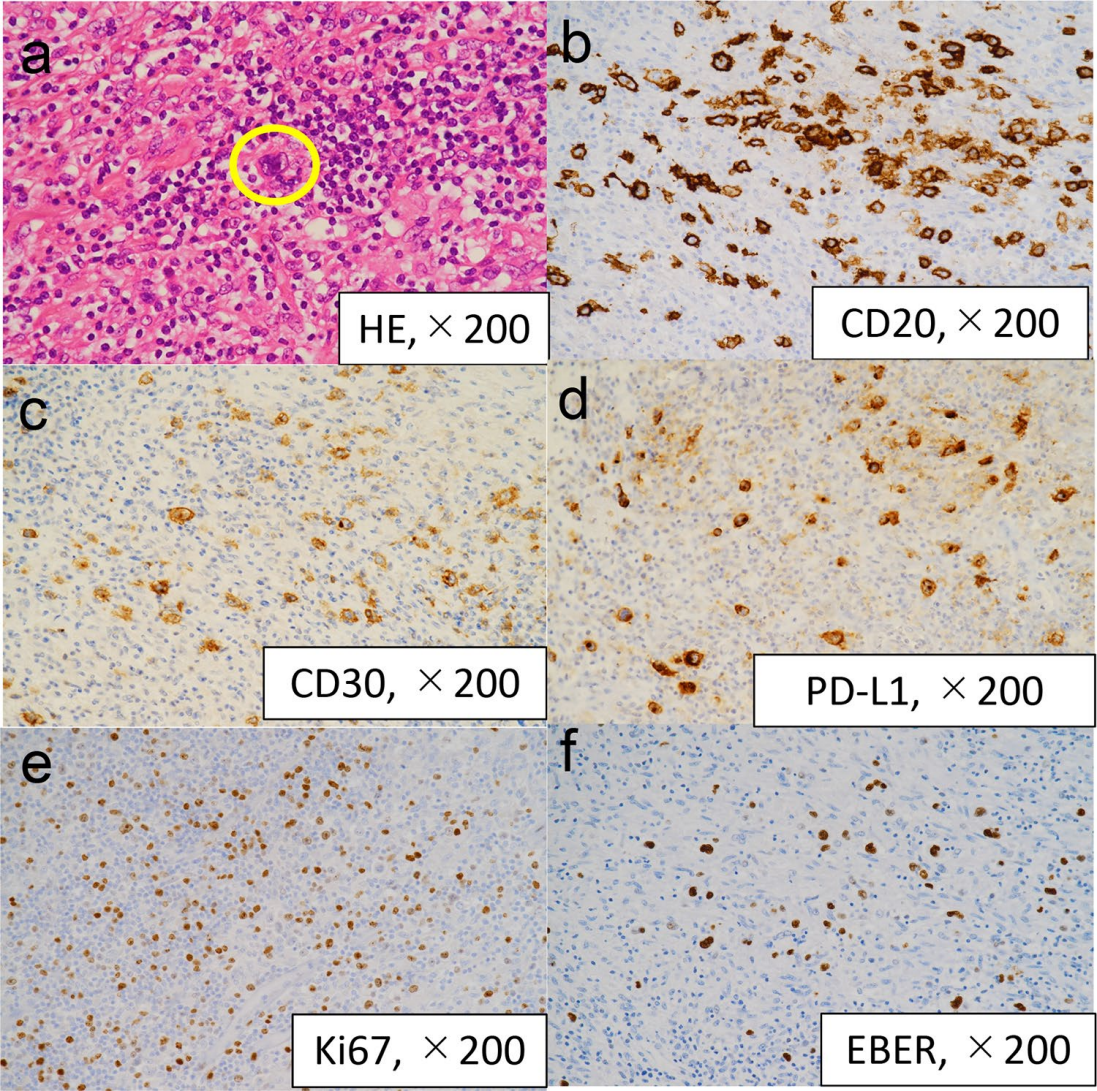
Histopathological, immunohistochemical, and in situ hybridization findings. **a** HE staining showing large atypical lymphoid cells with prominent nucleoli (Reed–Sternberg [RS] cells) within a background of extensive inflammation and immune cell infiltration. The RS cells are circled for clarity. **b** Immunohistochemical staining demonstrating that the RS cells were positive for CD20, indicating their B-cell origin. **c** Immunohistochemical staining for CD30 showing strong membranous positivity in the RS cells. CD30, a membrane protein belonging to the tumor necrosis factor receptor superfamily (TNFRSF8), is expressed on the surface of activated lymphocytes. **d** Immunohistochemical staining for PD-L1 showing strong membranous and cytoplasmic positivity in the RS cells, consistent with activation of the PD-1/PD-L1 immune evasion pathway characteristic of classic Hodgkin lymphoma. Background lymphocytes showed minimal or no PD-L1 expression. **e** Immunohistochemical staining for Ki-67 showing strong nuclear positivity in the RS cells, indicating high proliferative activity and active cell-cycle progression. Surrounding reactive lymphocytes showed only scattered or weak positivity. **f** In situ hybridization for EBER showing positive nuclear signals within the tumor cells, confirming Epstein–Barr virus infection. All images were captured at × 200 magnification. Abbreviations: HE, hematoxylin and eosin; PD-L1, programmed death-ligand 1; EBER, Epstein–Barr virus–encoded RNA

Histopathological examination of the sigmoid colon lesion revealed large atypical lymphoid cells with prominent nucleoli (Reed–Sternberg [RS] cells) within a background of extensive inflammation and immune cell infiltration (Fig. 5a). Immunohistochemical analysis demonstrated that the RS cells were positive for CD20 and CD30. CD20 positivity confirmed their B-cell origin, whereas strong CD30 expression, a feature of the tumor necrosis factor receptor superfamily, suggested the activation of downstream signaling pathways such as NF-κB and MAPK, which promote RS cell survival and proliferation (Fig. 5b, c) [8]. The RS cells also showed strong membranous and cytoplasmic expression of PD-L1, consistent with the activation of the PD-1/PD-L1 immune evasion pathway, which is characteristic of cHL (Fig. 5d). In addition, the RS cells exhibited intense nuclear staining for Ki-67, indicating high proliferative activity (Fig. 5e). In situ hybridization for EBV-encoded RNA (EBER) demonstrated positive nuclear signals within the RS cells, confirming EBV infection (Fig. 5f). RS cells and extensive inflammation and immune cell infiltration were also observed in the resected mesenteric lymph nodes. Based on these findings, the patient was diagnosed with EBV reactivation–associated cHL. According to the fifth edition of WHO classification, this case was categorized as lymphoma arising from immune deficiency and dysregulation [9].

**Fig. 6:**
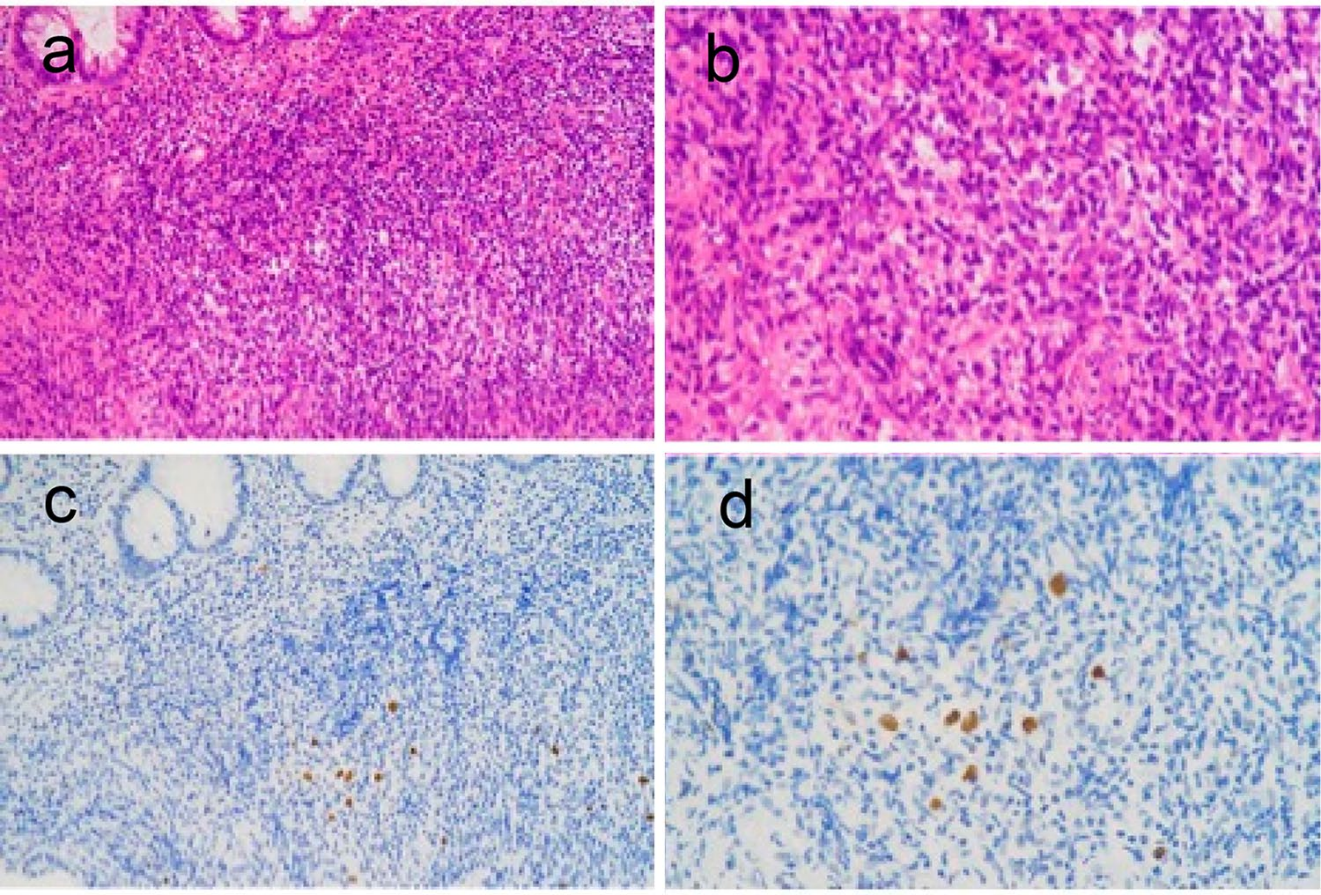
Histopathological findings of biopsy specimens obtained 18 months before surgery. **a** H&E staining at × 200 magnification shows inflammatory granulation tissue without overt cytological atypia. Reed–Sternberg–like cells were not identifiable on routine morphological assessment at that time. **b** H&E staining at × 400 magnification similarly demonstrates no definitive malignant features, and atypical large cells could not be recognized by conventional histology alone. **c** Epstein–Barr virus–encoded RNA (EBER) in situ hybridization at × 200 magnification reveals scattered EBER-positive large cells. **d** EBER in situ hybridization at × 400 magnification confirms the presence of EBER-positive large cells, which were relatively sparse compared with those observed in the surgical specimen

Retrospective re-evaluation demonstrated a small number of EBER-positive large cells on EBER in situ hybridization in both the biopsy specimen obtained approximately 18 months before surgery and the preoperative biopsy specimen (Fig. 6). Postoperative positron emission tomography (PET)–CT revealed no residual lesions with abnormal fluorodeoxyglucose uptake. Chemotherapy was recommended; however, the patient declined treatment and has remained recurrence-free for 10 months postoperatively.

## Discussion

With advances in UC treatment, the number of patients who can be managed long-term without surgery has increased. However, prolonged colonic mucosal inflammation is associated with an increased incidence of UC-associated neoplasia. In addition to neoplastic transformation, LPDs have also been reported in association with immunosuppressive therapy, including immunomodulators such as 6-mercaptopurine and azathioprine, as well as biologic agents such as anti–tumor necrosis factor-α antibodies [10].

A B-cell lymphoma characterized by a small number of malignant RS cells scattered within a background of extensive inflammation and immune cell infiltration, cHL exhibits a bimodal age distribution, with incidence peaks at approximately 20–30 and 50–70 years of age [11]. Among younger individuals, cHL is the most common type of lymphoma; however, it is relatively rare in older adults because of the increased incidence of other lymphoma subtypes, such as diffuse large B-cell, marginal zone, and follicular lymphomas [12]. EBV infection is considered to be causally associated with approximately 25–40% of cHL cases [11, 13]. This disease can also be associated with immunosuppressive conditions and has been reported in patients with human immunodeficiency virus infections and autoimmune diseases [11].

Vedolizumab is a gut-selective monoclonal antibody that binds the α4β7 integrin and inhibits its interaction with mucosal addressin cell adhesion molecule-1 (MAdCAM-1) expressed in gut-associated lymphoid tissues (GALT) and intestinal mucosa [14]. Through this mechanism, vedolizumab selectively antagonizes α4β7-mediated adhesion/extravasation of gut-homing lymphocytes—particularly memory/effector T-cell subsets—and thereby reduces intestinal lymphocyte trafficking without the broader systemic immune effects associated with nonselective α4β7 blockade [15, 16]. While this tissue-selective mechanism underlies its favorable safety profile, emerging human and experimental data suggest that α4β7 blockade may also modulate GALT, potentially attenuating local mucosal immune surveillance [17]. In susceptible hosts, such localized impairment of intestinal immune control could theoretically contribute to inadequate containment of oncogenic viruses such as Epstein–Barr virus. We emphasize that this remains a hypothesis, and causality cannot be inferred from a single case; however, the biology of α4β7–MAdCAM-1 trafficking provides a plausible framework for considering vedolizumab-associated disruption of intestinal immune surveillance as a contributing factor to EBV-associated lymphoma development.

In the present case, EBER in situ hybridization was positive, confirming that the tumor cells were infected with EBV. Immunomodulator therapy was discontinued eight years prior after only two weeks of treatment, and treatment with vedolizumab was initiated 31 months prior to the current presentation. Eighteen months prior, colonoscopy revealed early lesions later considered compatible with incipient cHL. Fifteen months prior, the patient was diagnosed with breast cancer and started aromatase inhibitor therapy, after which lymphoproliferative disease became clinically evident. These findings suggest that pathogenesis may have involved reactivation of latent EBV infection and subsequent proliferation of infected B cells under locally altered intestinal immune conditions associated with vedolizumab therapy. Similarly, a previous report described EBV-related mucosal ulceration occurring during vedolizumab treatment [18]. Vedolizumab selectively suppresses intestinal immune responses by inhibiting lymphocyte trafficking to the gut, which may create an environment prone to the development of focal intestinal immune deficiency and dysregulation-related lymphoma. To the best of our knowledge, this is the first reported case of cHL associated with vedolizumab therapy.

From a pathological perspective, the main differential diagnoses in the present case included EBV-associated mucocutaneous ulcer (EBV-MCU) and EBV-positive diffuse large B-cell lymphoma (DLBCL). According to the WHO Classification of Tumours of Haematolymphoid Tumours, 5th edition, EBV-MCU typically demonstrates broad EBER positivity across a wide spectrum of lymphoid cells of varying sizes [9]. In contrast, in cHL, EBER positivity is usually confined to a limited number of large atypical RS cells. In the present case, EBER in situ hybridization showed positivity restricted to scattered large atypical RS cells, whereas the majority of background cells were EBER-negative, a distribution pattern consistent with cHL rather than EBV-MCU. Furthermore, EBV-positive DLBCL is characterized by diffuse sheet-like proliferation of large atypical B cells. However, such a diffuse growth pattern was not observed in this case; instead, only rare large atypical cells were observed in the lesion. Taken together with the immunophenotypic findings, including CD30 positivity, CD20 expression in RS cells, and high PD-L1 expression, these findings supported the diagnosis of cHL.

Surgical resection may improve the prognosis of patients with localized malignant lymphoma when combined with chemotherapy; however, these findings are primarily based on cases of non-Hodgkin B-cell lymphoma, and the benefits for cHL remain unclear [19, 20]. Surgery alone is generally not considered a curative treatment for cHL, and standard management relies on systemic chemotherapy with or without radiotherapy [21]. In the present case, the sigmoid colon lymphoma was not diagnosed preoperatively. Surgery was considered appropriate because of the presence of high-grade dysplasia in the transverse colon and refractory bleeding, presumably caused by the sigmoid lesion, which did not respond to medical treatment. Other biological agents and immunomodulators that could suppress systemic antitumor immunity were not considered viable options because the patient was undergoing treatment for breast cancer. Ultimately, the patient declined the recommended chemotherapeutic regimen. In elderly patients with comorbidities, including concurrent malignancies, treatment decision-making for cHL often requires careful individualization, balancing potential therapeutic benefit against treatment-related toxicity and patient preference [22]. Although postoperative PET–CT revealed no significant lymph node involvement throughout the body, and the patient has remained recurrence-free for 10 months after surgery alone, this favorable short-term outcome should be interpreted with caution. Careful follow-up is warranted to monitor for potential disease progression.

The present case highlights the complexity of lymphoma development in elderly patients with UC. The occurrence of EBV-associated cHL in this patient should be interpreted within a multifactorial framework rather than being attributed to a single causative factor. Elderly-onset inflammatory bowel disease represents a growing clinical population, and age-related immune dysfunction is increasingly recognized as an important contributor to malignancy risk [23]. Immunosenescence may impair immune surveillance of latent viral infections, thereby facilitating EBV reactivation and lymphomagenesis. In addition, the patient had a history of breast cancer treated with an aromatase inhibitor. Although causal inference cannot be established, previous clinical reports and experimental studies have suggested that estrogen-deprived conditions may influence lymphoid proliferation [24, 25]. Aromatase inhibitor therapy may therefore represent a background factor potentially modulating lymphoma progression. Although the development of lymphoma in this case may have been influenced by multiple factors—including vedolizumab-related alterations in intestinal immunity, age-related immune dysfunction, and aromatase inhibitor therapy—clinicians should remain aware of the potential for intestinal lymphoma to occur during vedolizumab therapy.

In the present case, endoscopic biopsy of the sigmoid lesion performed prior to surgery did not reveal morphologically atypical lymphoid cells and was initially interpreted as granulation tissue. However, retrospective pathological re-evaluation demonstrated the presence of a relatively smaller number of EBER-positive large cells than were identified in the surgical specimen. Routine histological evaluation alone may be insufficient to detect early-stage EBV-associated lymphoma. Therefore, when lymphoma is clinically suspected—particularly in patients with UC receiving biologic agents or immunomodulatory therapy—EBER in situ hybridization should be actively considered, even when initial biopsy specimens lack clear morphological features of malignancy. EBER staining may serve as a valuable adjunctive tool for the early detection of EBV-associated lymphoproliferative disorders and facilitate timely diagnosis.

Gastrointestinal lymphomas most commonly present as ulcerative lesions with raised, ear-like margins, whereas other morphological patterns are less frequent. According to the endoscopic classification proposed by Kanno et al., the lesion observed in the present patient approximately 18 months before surgery was consistent with the ulcerative type, characterized by shallow ulceration with only mild mural thickening [26]. In contrast, the lesion at the time of surgery demonstrated circumferential and markedly thickened mural involvement, which did not clearly fit into any single category of this classification system, although it most closely resembled a diffuse infiltrative pattern. Such a morphology is considered extremely uncommon and may reflect an advanced stage of disease progression. Histological examination of biopsy specimens obtained both 18 months before surgery and at the preoperative endoscopy demonstrated inflammatory granulation tissue; however, retrospective pathological re-evaluation revealed scattered EBER-positive large cells in both biopsy specimens, indicating that EBV-driven lymphoproliferative changes were already present at an earlier stage, even when the lesion showed a morphology closer to the typical ulcerative pattern. In cHL, neoplastic Reed–Sternberg cells are typically sparse and embedded within a prominent inflammatory background, making them particularly difficult to distinguish from reactive inflammatory cells on routine hematoxylin–eosin staining. These findings suggest that reliance on routine histology alone may delay the diagnosis of EBV-associated cHL. Therefore, when gastrointestinal lymphoma is clinically suspected, close communication between clinicians and pathologists is essential. Clinicians should clearly convey their clinical suspicion and proactively discuss the indication for additional ancillary studies, including EBER in situ hybridization, to facilitate timely and accurate diagnosis.

In conclusion, EBV-associated malignant lymphomas, including cHL, may develop in patients with UC receiving vedolizumab therapy; however, this report represents a single case, and a direct causal relationship between vedolizumab and EBV reactivation cannot be established. Despite this limitation, clinicians should maintain a high index of suspicion when lesions suggestive of neoplastic change, such as intestinal wall thickening with ulceration, are observed during vedolizumab treatment and should consider early histopathological evaluation with malignant lymphoma in mind to ensure timely and appropriate clinical management.

## References

[1] Silverberg MS, Satsangi J, Ahmad T, et al. Toward an integrated clinical, molecular and serological classification of inflammatory bowel disease: report of a Working Party of the 2005 Montreal World Congress of Gastroenterology. Can J Gastroenterol. 2005;19(Suppl A):5A-36A. 16151544 10.1155/2005/269076

[2] GBD 2017 Inflammatory Bowel Disease Collaborators. The global, regional, and national burden of inflammatory bowel disease in 195 countries and territories, 1990–2017: a systematic analysis for the Global Burden of Disease Study 2017. Lancet Gastroenterol Hepatol. 2020;5(1):17–30. 10.1016/S2468-1253(19)30333-4 PMC7026709 31648971

[3] Ananthakrishnan AN. Debate session: so what causes inflammatory bowel disease? It’s all in the environment. J Gastroenterol Hepatol. 2018;33(Suppl 3):24. 30187567 10.1111/jgh.14429

[4] Quan T, Li R, Gao T. The intestinal macrophage-intestinal stem cell axis in inflammatory bowel diseases: from pathogenesis to therapy. Int J Mol Sci. 2025;26(7):2855. 40243444 10.3390/ijms26072855 PMC11988290

[5] Yang L, Li H, Tang M, et al. Circular RNAs in inflammatory bowel disease: a review of mechanisms, biomarkers and therapeutic potential. Front Immunol. 2025;16:1540768. 40342413 10.3389/fimmu.2025.1540768 PMC12058709

[6] Nissen LH, Nagtegaal ID, de Jong DJ, et al. Epstein-Barr virus in inflammatory bowel disease: the spectrum of intestinal lymphoproliferative disorders. J Crohns Colitis. 2015;9(5):398–403. 25740811 10.1093/ecco-jcc/jjv040

[7] Feagan BG, Rutgeerts P, Sands BE, et al. Vedolizumab as induction and maintenance therapy for ulcerative colitis. N Engl J Med. 2013;369(8):699–710. 23964932 10.1056/NEJMoa1215734

[8] Sadaf H, Ambroziak M, Binkowski R, et al. New molecular targets in Hodgkin and Reed–Sternberg cells. Front Immunol. 2023;14:1155468. 37266436 10.3389/fimmu.2023.1155468 PMC10230546

[9] Swerdlow SH, Campo E, Harris NL, et al. Hodgkin lymphoma. In: IARC, ed. WHO Classification of Tumours: Haematolymphoid Tumours. 5th ed. Lyon: IARC Press; 2022.

[10] Sato Y. Highlights: focus on immunodeficiency-associated lymphoproliferative disorders. J Clin Exp Hematop. 2019;59(2):46–7. 31257344 10.3960/jslrt.19020 PMC6661960

[11] Brice P, de Kerviler E, Friedberg JW. Classical Hodgkin lymphoma. Lancet. 2021;398(10310):1518–27. 33493434 10.1016/S0140-6736(20)32207-8

[12] Smith A, Crouch S, Lax S, et al. Lymphoma incidence, survival and prevalence 2004–2014: sub-type analyses from the UK’s haematological malignancy research network. Br J Cancer. 2015;112(9):1575–84. 25867256 10.1038/bjc.2015.94 PMC4453686

[13] Urayama KY, Jarrett RF, Hjalgrim H, et al. Genome-wide association study of classical Hodgkin lymphoma and Epstein-Barr virus status-defined subgroups. J Natl Cancer Inst. 2012;104(3):240–53. 22286212 10.1093/jnci/djr516 PMC3274508

[14] Soler D, Chapman T, Yang LL, et al. The binding specificity and selective antagonism of vedolizumab, an anti-alpha4beta7 integrin therapeutic antibody in development for inflammatory bowel diseases. J Pharmacol Exp Ther. 2009;330(3):864–75. 19509315 10.1124/jpet.109.153973

[15] Arijs I, De Hertogh G, Machiels K, et al. Effect of vedolizumab (anti-α4β7-integrin) therapy on histological healing and mucosal gene expression in patients with UC. Gut. 2018;67(1):43–52. 27802155 10.1136/gutjnl-2016-312293

[16] Luzentales-Simpson M, Pang YCF, Zhang A, et al. Vedolizumab: potential mechanisms of action for reducing pathological inflammation in inflammatory bowel diseases. Front Cell Dev Biol. 2021;9:612830. 33614645 10.3389/fcell.2021.612830 PMC7887288

[17] Canales-Herrerias P, Uzzan M, Seki A, et al. Gut-associated lymphoid tissue attrition associates with response to anti-α4β7 therapy in ulcerative colitis. Sci Immunol. 2024;9(94):eadg7549. 38640252 10.1126/sciimmunol.adg7549 PMC11140591

[18] Fernandes C, Catinis C, Anyanwu P, et al. Epstein-Barr virus associated ulcers in a patient with ulcerative colitis taking vedolizumab: a case report. Am J Gastroenterol. 2023;118(10S):S2151.

[19] Zhang C, Zhang X, Liu Z, et al. The impact of surgery on long-term survival of patients with primary intestinal non-Hodgkin lymphomas based on SEER database. Sci Rep. 2021;11(1):23047. 34845308 10.1038/s41598-021-02597-1 PMC8630038

[20] Hong YW, Kuo IM, Liu YY, et al. The role of surgical management in primary small bowel lymphoma: a single-center experience. Eur J Surg Oncol. 2017;43(10):1886–93. 28751057 10.1016/j.ejso.2017.06.016

[21] Eichenauer DA, Aleman BMP, André M, et al. Hodgkin lymphoma: ESMO Clinical Practice Guidelines. Ann Oncol. 2018;29(Suppl 4):iv19–iv29. 10.1093/annonc/mdy080 29796651

[22] Böll B, Görgen H. The treatment of older Hodgkin lymphoma patients. Br J Haematol. 2019;184(1):82–92. 30407626 10.1111/bjh.15652

[23] Akiyama S, Ito Y, Shiroyama M, et al. Increasing age at diagnosis raises malignancy risk and aminosalicylate intolerance influences therapeutic strategies in ulcerative colitis: a multicenter I-BRITE cohort study. J Gastroenterol. 2025;60:1259–71. 40627077 10.1007/s00535-025-02279-z

[24] Kitagawa Y, Nassiri M, Mesa H, et al. Possible role of anastrozole-induced hormonal alterations in pathogenesis of mammary apocrine carcinoma and follicular lymphoma: a case report and review of the literature. J Med Case Rep. 2025;19:465. 41024216 10.1186/s13256-025-05553-z PMC12482693

[25] Talaber G, Yakimchuk K, Guan J, et al. Inhibition of estrogen biosynthesis enhances lymphoma growth in mice. Oncotarget. 2016;7(15):20724–38. 10.18632/oncotarget.7843 PMC4991487 26943574

[26] Kanno T, Katano T, Shimura T, et al. Characteristic endoscopic findings of gastrointestinal malignant lymphomas other than mucosa-associated lymphoid tissue lymphoma. Acta Gastroenterol Belg. 2022;85(3):477–83. 35770281 10.51821/85.3.9712

